# Item generation for a tooth wear-specific patient-reported outcome measure

**DOI:** 10.1186/s12955-025-02472-x

**Published:** 2026-03-17

**Authors:** Melissa Sin, Richard Holliday, Helen Rogers, Rijula Karanjkar

**Affiliations:** 1 School of Dental Sciences, Faculty of Medical Sciences, Newcastle University, Newcastle upon Tyne, NE2 4BW UK; 2 Newcastle Upon Tyne Hospitals NHS Foundation Trust, Newcastle upon Tyne, UK

**Keywords:** Tooth wear (MESH); patient reported outcome measure (MESH), Quality of life (MESH), Oral health related quality of life

## Abstract

**Introduction:**

The effective management of tooth wear is one of the biggest challenges faced in modern dentistry. Tooth wear can significantly impact oral health-related quality of life, yet there is no existing condition-specific patient-reported outcome measure (PROM) available to accurately capture and comprehensively measure this impact. This study aims to qualitatively explore patient perspectives of tooth wear to generate items for a condition-specific PROM.

**Methods:**

Adults referred to Newcastle Dental Hospital for management of diagnosed tooth wear were invited to participate in a semi-structured qualitative interview. Participants were purposively selected from a range of ages, genders, with varied tooth wear aetiology and severity, and at different stages of treatment. Recruitment continued until purposive sampling requirements had been met, and data saturation was reached. Thematic analysis was undertaken independently by two researchers, prior to discussion to reach agreement on the identified themes.

**Results:**

Seventeen participants with a range of tooth wear aetiologies, severity and at different stages of treatment were interviewed. Thematic analysis produced three broad themes including psychosocial impact of tooth wear, effect on function and knowledge and understanding of tooth wear.

**Conclusions:**

This study demonstrates the broad impacts that tooth wear can have on patients’ oral health-related quality of life, and has generated a wealth of items to inform generation of a condition-specific PROM. Understanding how tooth wear can impact on oral health-quality of life from a patients perspective is a critical first step in generation of a condition-specific patient-reported outcome measure.

**Supplementary Information:**

The online version contains supplementary material available at 10.1186/s12955-025-02472-x.

## Introduction

Tooth wear is a multifactorial dental condition, which can lead to the irreversible loss of mineralised tooth substance, due to processes other than dental caries [1, 2]. 15% of adults in the UK suffer from moderate tooth wear and 2% suffer from severe disease [2]. Tooth wear can be erosive in nature or mechanical in origin, with intrinsic and extrinsic forms. It often can be attributed to a combination of these, which can make diagnosis and management difficult [3]. Tooth wear can have significant impacts on an individual’s quality of life, with greater psychological discomfort and disability affecting those with more severe tooth wear [4–7]. Whilst many patients may experience no symptoms, dental care is often sought due to sensitivity, difficulty eating, aesthetics and noticeable loss of tooth structure [2]. Wider impacts on daily life have also been reported, such as feeling embarrassed and self-conscious, and experiences of social disability and dissatisfaction of living [4]. The aetiological factors that cause tooth wear can have wider impacts on an individual’s life, in addition to leading to the symptoms of tooth wear. This includes stress- or anxiety-induced clenching, grinding or compulsive tooth brushing. The involvement of dental care professionals has been recommended within the literature to facilitate the identification and improvement of medical and mental health issues, to tackle the aetiological factors of tooth wear within primary care [8]. The management of tooth wear varies, depending on its severity, ranging from preventative strategies, to full mouth rehabilitation. This is usually influenced by patient motivations, stability of the wear and financial circumstances [9]. Assessing treatment outcomes for patients with tooth wear is an important step in monitoring and improving the quality of care and service delivery. This is often assessed through use of clinical outcome measures such as restoration quality or longevity. However, several aspects of treatment outcomes are best captured by patient-related outcome measures (PROMs) [10]. They report patients’ perspectives with their well-being, functioning, symptoms and experiences with treatment. Several generic oral health related quality of life (OHRQoL) questionnaires are available (e.g. Oral Health Impact Profile (OHIP) and Dental Impacts on Daily Living (DIDL)) and have been used to capture the patient-reported impacts of tooth wear on OHRQoL in previous research [5–7, 11–13]. Other self-reporting instruments derived from prosthodontic patients have been described such as the Orofacial Esthetic Scale (OES), however, this is based primarily on aesthetics only, albeit a common complaint from tooth wear patients [14]. 

The generic instruments are designed to capture all impacts of oral health conditions, hence whilst they have a role in both clinical care and research, they may also lack the sensitivity to capture more specific impacts of conditions such as tooth wear [15]. The literature suggests that condition-specific instruments are more responsive to changes in health-related quality of life [15]. Moreover, at the time when these generic instruments were developed, there was less emphasis placed on research involving patients and the public compared to the present day and indeed shared clinical decision making compared to the present day [13]. This highlights a limitation of many generic instruments, as optimal patient involvement at every stage of instrument development can ensure the relevance of outcomes and comprehensibility of a measure [16, 17]. 

There is currently no existing condition-specific PROM available for tooth wear, as shown in a recent literature review [18]. A tooth wear-specific PROM could have varied uses in both research and clinical care. For example, the impact of tooth wear and its management could be captured alongside clinical outcomes and this data could help support commissioning of services and validating the benefit of these services in the longer term.

This study aims to undertake the first step in developing a condition-specific PROM for tooth wear, known as item generation [19]. This involves qualitative exploration of patient perspectives of tooth wear to identify the impacts and experiences of individuals diagnosed with this condition.

## Methods

This study was conducted using semi-structured interviews to generate rich data which were analysed using inductive thematic analysis [20]. Ethical approval for this study was granted by Newcastle University Faculty of Medical Sciences Research Ethics Committee reference number 20/LO/0190.

This manuscript has been written in accordance with the consolidated criteria for reporting qualitative research checklist (COREQ) [21]. 

### Participants

Patients with diagnosed tooth wear who had been referred into Newcastle Dental Hospital for assessment of tooth wear were invited to take part in this study. The inclusion criteria were patients who had been assessed, were undergoing treatment or had completed a full course of treatment for the management of tooth wear within Newcastle Dental Hospital. Exclusion criteria included patients with tooth wear due to congenital dental defects and patients undergoing treatment for head and neck oncology.

### Sampling

Participants were purposively sampled to represent a range of tooth wear aetiologies and stages of tooth wear management. Data on aetiology and tooth wear severity staging (mild, moderate or severe) were derived as documented diagnoses from patient records. The staging of mild, moderate and severe was based on the clinical adaptation of the Smith and Knight toothwear index, which was used as part of clinical assessment at Newcastle Dental Hospital at the time [22]. Participants were recruited and interviewed until no new ideas were identified and it was agreed that data saturation had been reached amongst the research team.

### Participant recruitment

Prospective participants were contacted by their normal clinical care team. If expression of interest was given, they were provided with an information sheet and consent form either in person or by mail. Participants were advised that they would receive a £15 shopping voucher on completion of the interview as a token of appreciation for participation. Additionally, participants travelling to Newcastle Dental Hospital for an interview were given an additional £10 shopping voucher as reimbursement for travel expenses. Once the consent form had been completed, the participant was invited for an interview. Participants were able to withdraw from the study at any time.

### Interviews

Qualitative interviews were conducted by MS either in person at Newcastle Dental Hospital’s Dental Clinical Research Facility or through Zoom (Zoom Video Communications Inc, San Jose, CA, USA) due to the presence of the COVID-19 pandemic. The study was explained to participants verbally prior to the interview and consent was confirmed. A topic guide was used to structure the interviews, which explored participants’ experience of tooth wear pre-treatment, during treatment and its impact on their lives (Supplementary files). This was designed with active involvement of a member of the public, to ensure the language used and interview format were appropriate. The topic guide was piloted and developed iteratively as the interviews progressed. The first three interviews were reviewed amongst the team to help develop the topic guide and interviewing technique. Interviews were transcribed verbatim by a third party and reviewed by the research team. Transcripts were returned to a sample of participants following their interview for their feedback and to check accuracy, and an opportunity was given for correction.

### Reflexivity

The lead interviewer was a female junior member of the hospital dental team, and was new to qualitative research. She was supported by an early career researcher and clinician with a Masters’ degree (RK) and two experienced clinical academics with PhDs (RH and HR) and particular expertise in qualitative research. Bespoke training in qualitative interviewing was provided and practice interviews were conducted with feedback.

Three members of the research team (MS, RK and RH) had a vested interest in the topic as their clinical roles involved provision of restorative care for adults with tooth wear. The potential for future research to be conducted using a condition-specific PROM was a particular draw of this study for these members of the team. The fourth member of the research team (HR) was a paediatric dentist and hence had minimal experience in caring for adults with tooth wear.

The lead interviewer (MS) was not part of the participants usual care team at NDH and hence was not known to any participants. They were introduced to participants as a researcher, and wore non-clinical clothing to avoid introducing any power influence.

### Analysis

Coding was conducted on the interview transcripts by MS and RK, using NVivo version 12 software (QSR International PSY Ltd., Melbourne, Australia) to organise the data. Both researchers discussed their analysis to reach agreement on themes using a constant comparative approach, until consensus on the meaning of the data was reached [20]. The data were then organised into themes after discussion of the codes with the full research team. This was the focus of the narrative thematic analysis which aligns with the first stage of the seven-step process in developing a PROM proposed by Guyatt and colleagues [19]. Potential items for a PROM were identified, which will be further refined through the item reduction stage and then validated in future research. Patient experience and post-treatment experience data will be reported on as part of a separate publication describing domains relevant for a PREM in tooth wear. All narrative analyses were reviewed by the full research team and then summarised for this paper.

## Results

There were 19 potential participants and 17 were interviewed. Two participants did not return the consent form and therefore were excluded from interviews. All participants were interviewed by MS, who had not treated the respondents as patients. No repeat interviews were conducted. Table 1 shows details of participant demographics.

**Table 1:** Participant demographics

Participant demographics	*N*/17
**Gender**	
Female	4 (23.5%)
Male	13 (76.5%)
**Age**	
16–44	1 (5.9%)
45–54	4 (23.5%)
55–64	6 (35.3%)
65 and over	6 (35.3%)
**Aetiology**	
Parafunction	5 (29.4%)
Intrinsic gastric	5 (29.4%)
Dietary extrinsic	3 (17.6%)
Eating disorder	2 (11.8%)
Unknown aetiology	1 (5.9%)
Combination	1 (5.9%)
**Stage of treatment**	
Assessment	5 (29.4%)
Mid-treatment	3 (17.6%)
Post treatment	9 (52.9%)

Analysis of qualitative data resulted in three overarching themes which are outlined below. Table 2 shows the overarching themes, subthemes and coding tree.

**Table 2:** Themes and coding tree

Themes	Subthemes	Codes
Psychological impact	Appearance	Change my face shape; Discolouration; Desire for bigger teeth; Desire to maintain health of teeth; Chipped; Jagged; Flat; Thin; Translucency; Cracks; Uneven; Not normal; Short teeth; Unsightly; Yellow; Stained; Staining; Small stumps; Dislike; Feeling old; Sunken cheeks; Asymmetrical; Breaking; Non-existent; Square teeth; Overclosure; Ridges; Horrible; Transparent; Crooked; Flat; Tiny; Blunt; Shearing; Teeth half the size
	Social interaction	Smiling with lips; Mask wearing; Hiding teeth; Reclusive; Fear of judgement; Smile differently; Reserved; Hiding true personality; Don’t want to open mouth; Anti-social; Covering face due to tooth wear; Big effect on life; Self-conscious; Hopelessness; Despair; Embarrassment; Effect on speech and demeanour; Confidence issues; Affecting personality; Low confidence; Self-blame; Self-hatred; Shame; “shrinking violet”; Shying away from photographs; Tooth wear preventing smiling; Tooth wear affecting mental health; Insecure; Embarrassment with dentures; Tooth wear changing personality; Affecting ability to speak
Function	Eating	Changes to occlusion; Limiting eating to certain teeth; Food stuck in teeth; Limiting eating certain foods; Decreased ability to bite; Difficulty eating chewy and hard foods; Decreased pleasure when eating; Struggling eating food; Conscious eating; Slow eating; Smaller bites; Abnormal eating habits; Affecting types of food eaten; Teeth breaking; Teeth snapping; Teeth crumbling
	Trauma during function	Gum damage; Soft tissue trauma; Trauma to tongue
	Sensitivity during function	Sensitivity; Rushing eating due to discomfort; Use of sensitive toothpaste; Sensitivity to cold; Sensitivity breathing in air; Sensitivity when brushing
Knowledge and information regarding tooth wear		Understanding of dietary acid as aetiology; “Soft teeth”; Difficulty in determining tooth wear aetiology; Tooth wear caused by reflux, realisation by patient; Thorough exam; Dentist monitoring tooth wear; Patient aware of tooth wear for several years; Dentist noticing first; Tooth grinding; Internet research; Missed explanations regarding aetiology; Ownership of habits; Perception of tooth wear as normal; Unclear understanding of aetiology post assessment; Understands aetiology; Recognising behaviours relating to aetiology; Difficulty managing diet; Confusion about dietary causes of wear; Wear not always identified to patient; Ill-informed about oral care; Varied understanding of wear aetiology

### Psychosocial impact

Tooth wear was identified to have significant impacts on participant’s perception of their appearance, their tendency to smile, their confidence and social interaction with others.

#### Appearance

Participant descriptions of their appearance were multifaceted. Descriptors used for worn teeth included “small”, chipped, flat, thin, uneven, stumps, broken, jagged, square, non-existent and tiny.

they were… *like* flat and straight, but jagged. (had) chips missing out of them. And …very thin. And … a little bit shorter as well.” (Participant 10: 39-year-old male, extrinsic dietary aetiology).

*They were just breaking and cracking and looking thin.* (Participant 3: 46-year-old female, intrinsic gastric aetiology)

The colour of worn teeth was highlighted by participants, negative connotations of yellower teeth being unsightly.

*Just really really conscious of my—particularly my bottom—my bottom teeth wearing away and exposing you know, the yellow part of the tooth. Um, which was unsightly. I had really bad stained teeth as well*. (Participant 17: 48-year-old male, parafunctional aetiology)

#### Social interaction

Tooth wear had a negative impact on participant’s self-esteem and confidence.

*So, they were yellow and it made a big difference in my self-confidence. It just hammered it you know?* (Participant 16: 63-year-old male, intrinsic gastric aetiology)

Participants expressed a fear of judgement from others regarding their tooth wear condition and feelings of embarrassment because they let their tooth wear condition progress and associated this with ineptness and dirtiness.

*Because you think yourself*,* “how did I manage to get into that situation where that went that far?” Other people’s obviously going to be thinking that. “Well*,* does he not care?” You know. “He seems to be clean and tidy*,* he seems to be this that and the other. He seems reasonably intelligent*,* so why has he done that?* (Participant 11: 67-year-old male, parafunction aetiology)

*I can’t smile without showing my teeth. And so*,* I was ashamed of my teeth and I thought people would look at my teeth and judge me.* (Participant 4: 61-year-old female, intrinsic gastric aetiology).

In addition to fearing judgement, participants also felt shame. They felt this affected their mental health and led to feelings of self-hatred and resultant neglect due to the appearance of their teeth.

*Yeah*,* I was worried because I was ashamed of the way my teeth looked because I’d let them get like that. (*Participant 4: 61-year-old female, intrinsic gastric aetiology)

This led to participants hiding the appearance of their teeth. This included wearing surgical face masks or changing their appearance to make their mouth less noticeable. When interacting when other people, participants described covering their face when talking, mumbling or adjusting the shape of their mouth when speaking to avoid showing people their teeth. For some, the Covid-19 pandemic allowed a sense of freedom in socialising with a mask, as covering your face was socially acceptable.

*I don’t mind wearing masks. It hides the teeth. But*,* it really affected me mental health like really bad. Me pride and me*,* you know*,* just everything about it.* (Participant 7: 52-year-old male, extrinsic dietary aetiology)

*Well*,* it wasn’t so bad the past two years because I wear a mask all the time [context of pandemic] …But I suppose once we come out of the masks then yeah*,* it would affect me.* (Participant 4: 61-year-old female, intrinsic gastric aetiology)

Others reported finding alternative strategies to make their teeth less noticeable in interpersonal interactions. Sometimes, these strategies impacted their ability to communicate with clarity, further impacting confidence.

*Um*,* I try to have a beard on to try and hide it. You know what I mean?” (Participant 7: 52-year-old male*,* extrinsic dietary aetiology*)

*That shot my confidence well down. And because of that I used to speak*,* and sometimes people used to say*,* “You’re mumbling”*,* because I wouldn’t speak properly. I wouldn’t open my mouth because I was embarrassed of the teeth. So in that respect that was a big thing.* (Participant 16: 63-year-old male, intrinsic gastric aetiology)

Participants actively avoided showing their teeth in social situations, through either changing their style of smiling or avoiding smiling altogether to avoid judgement from others.

*You kind of learn to smile a little bit differently…when I used to laugh*,* I used to throw me head back and you know like*,* “Ahhh”. That got a bit more reserved*,* just so people didn’t see my teeth as much. Or I’d kind of put my—like pretend I was rubbing my nose or scratching my eye or*,* you know*,* something just to sort of cover my mouth a little bit.* (Participant 13: 47-year-old female, eating disorder aetiology)

*If I smile it’s with me mouth shut… it’s massively affected me life this*. (Participant 7: 52-year-old male, extrinsic dietary aetiology).

Negative comments from family members provided reminders for participants regarding their dental condition and acted as drivers or prompts for them to seek treatment.

*It was mainly my wife that pointed it out. She just said that they looked a bit*,* horrible…I mean I noticed it myself but yeah*,* she reminded me of it.* (Participant 10: 39-year-old male, extrinsic dietary aetiology)

The impact of the appearance of their teeth on their confidence lead to some withdrawal from normal social circles. This included not only face-to-face meetings but virtual encounters.

*And looking in the mirror every day when I’m cleaning my teeth*,* I’m noticing that that tooth is getting smaller and smaller and smaller. And of course*,* in the days of Teams meetings and Zoom*,* you’re looking at yourself more… And I’m really conscious that*,* you know*,* sometimes I turn my camera off because I don’t like the way my teeth are getting* (Participant 17: 48-year-old male, parafunctional aetiology)

*And I felt like I was hiding a lot still because of my teeth. And not seeing*,* you know*,* friends and so basically I was embarrassed. Because they were visually awful to look at.* (Participant 13: 47-year-old female, eating disorder aetiology)

This reduced self-care and pride, due to their perception of poor appearance reduced their motivation to maintain good oral health.

*…sometimes I felt disheartened and I thought*,* what the hell am I cleaning these stumps for? […] to be honest with you*,* I thought I would be better off having all my teeth taken out— (*Participant 16: 63-year-old male, intrinsic gastric aetiology)

Overall, the psychosocial impact of tooth wear was clear in the extent to which it influenced self-confidence, altered behaviours and intrapersonal relationships.

### Function

Participants had varied experiences regarding the effect of tooth wear on the function of their teeth and daily routines.

#### Eating

Participants described adapting their method of eating by chewing food in certain parts of their mouth or using utensils in a certain way. This led to more conscious eating which meant participants felt that they ate slowly. Participants were aware that this may not be considered normal and could be awkward in social situations.

*I can eat slower*,* I can put me food in me mouth slower. I can start moving me mouth round really gently and slowly with whatever meal—* (Participant 11: 67-year-old male, parafunction aetiology)

*I knew I wasn’t eating or chewing the way that*,* sort of normal people as it were would chew their food.* (Participant 14: 61-year-old male, eating disorder aetiology)

*Yeah*,* the front teeth had suffered quite a bit of wear and it was a struggle to actually take a bite out of a sandwich so it was something—I just used to eat everything I could with a knife and fork.* (Participant 5: 66-year-old male, extrinsic dietary aetiology)

Participants also felt that their front teeth were being overused, especially with chewing foods such as meat.

*I use the—my front teeth a lot because I’m missing quite a few of the incisors and cutters on the side teeth. So they’re put to a lot of use.* (Participant 1: 67-year-old male, parafunction aetiology)

Some did not change their food habits but persevered with discomfort and pain to maintain their usual diet.

*I didn’t really change me food habits. I just struggled through it. I mean I love crusty bread. … it would dig into me gums.* (Participant 14: 61-year-old male, eating disorder aetiology)

Some reported adaptation to these changes when eating, particularly using their front teeth to maintain adequate function.

*Yeah*,* I didn’t—it didn’t really bother me that much*,* because I suppose I just got used to the fact that I didn’t have the molars and I was using my front teeth to actually chew meat and other*,* heavy textured foodstuffs—* (Participant 1: 67-year-old male, parafunction aetiology)

In addition, participant’s experienced difficulty eating certain foods due to their limited ability to function with their dentition. This included a diminished ability to eat hard foods or excessive food trapping. This often prevented them from enjoying foods they loved and forced them to revert to softer diets.

*Well what it was was basically was getting to a point where certain days I couldn’t eat—I couldn’t you know*,* like if you had like say a chop or something*,* you couldn’t chew it properly or anything* (Participant 6: 63-year-old male, intrinsic gastric aetiology)

*And there are several different kinds of food where*,* you know I’ve said*,* “Oh no*,* I can’t eat that”. I try*,* but I think*,* “Oh no*,* I can’t”. It’s just impossible.* (Participant 8: 75-year-old female, parafunction aetiology)

This in turn led to frustration and dissatisfaction, reducing the joy of having a meal, as they found themselves rushing their meals.

*And I was just—I would rush the food or the drink to get it*,* done and dusted*,* so that it wouldn’t prolong it.* (Participant 16: 63-year-old male, intrinsic gastric aetiology)

The process of breaking either gradually or when eating food was often described with varying experiences with regards to the structure of their teeth changing over time.

*If I ate something hard*,* bit into it hard*,* occasionally they’d snap or break.* (Participant 3: 46-year-old female, intrinsic gastric aetiology)

*they’re just crumbling (teeth) .and there’s bits where it’s coming off* (Participant 6: 63-year-old male, intrinsic gastric aetiology)

#### Trauma during function

Participants described accidentally biting their soft tissues during mealtimes, causing trauma, despite conscious effort to adapt eating habits. Other hard foodstuffs would be pushed into the gums due to lack of functioning teeth, causing discomfort. This led to cautious eating to avoid bleeding and trauma.

*I did used to get a lot of cuts in me mouth. I used to catch me tongue and catch the side of me cheek*,* and take chunks out of it. Sort of to the point where you know*,* it was sometimes quite bad and bleeding.* (Participant 14: 61-year-old male, eating disorder aetiology)

#### Sensitivity during function

Other experiences important to participants were feeling of sensitivity to cold, breathing in air and brushing their teeth. Participants used emotive language regarding their sensitivity which emphasised the burden it had on their daily lives.

*I’ve got to wait (for food to be room temperature)… anything cold just absolutely kills us* (Participant 7: 52-year-old male, extrinsic dietary aetiology)

*Um*,* yeah. Just the usual. Hot/cold*,* to start with*,* but you know it*,* at the end it was breathing in air? You know*,* breathing with my mouth open even just the air going in*,* would sort of*,* they would be sensitive. Especially if it was cold*,* and you’re outside.”* (Participant 13: 47-year-old female, eating disorder aetiology)

This sensitivity also posed a barrier to effective oral hygiene habits.

*Towards the end it was—it would hurt to brush my teeth.* (Participant 13: 47-year-old female, eating disorder aetiology)

Some patients used sensitive toothpaste to alleviate this with mixed opinions on its effectiveness. Sensitive toothpastes did not always help manage symptoms.

*And I do—well*,* I say I do—I did*,* used to use Sensodyne toothpaste. But that’s—didn’t really take the edge of it to be honest.* (Participant 14: 61-year-old male, eating disorder aetiology)

Participants showed resilience to symptoms they experienced but this often meant putting up with discomfort.

*I tried not to have it limit of and—I’m a stubborn person so I like to fight against whatever’s trying to make it awkward for me. In all walks of life. Including my teeth. And so*,* I would put up with it. (‘discomfort*,* and with hot or cold drinks*,* then you would have pain you know?’)* (Participant 16: 63-year-old male, intrinsic gastric aetiology)

In contrast, other participants had little experience of sensitivity, with no impacts on their day-to-day life and some considered sensitivity to be a normal part of ageing.

*Um*,* I had no problems*,* I didn’t have any—they weren’t sensitive at all—I could eat you know*,* cold drinks or any—I could eat apples*,* I could eat fruit*,* anything*,* no problem at all. I had no problems whatsoever—no side effects*,* nothing at all. Nothing at all.* (Participant 15: 67-year-old male, unknown aetiology)

*Well*,* I almost always used Sensodyne as a toothpaste. But*,* no I can’t say I was suffering any sensitivity or—not more than I would have guessed for a person of my age.* (Participant 5: 66-year-old male, extrinsic dietary aetiology)

Functional issues were not universal, and some participants were able to adapt and eat without concern. However, individuals appear to have different degrees of tolerance and adaptability.

*To be fair I didn’t really avoid food*,* I just kind of got on with it really.* (Participant 2: 55-year-old male, parafunction aetiology).

When asked about the ability to eat: *“Nothing at all. No*,* I had no problems with that at all. No*,* nothing at all*,* no.”* (Participant 15: 67-year-old male, unknown aetiology).

### Knowledge and information regarding tooth wear

There was varied understanding amongst participants regarding the cause of their tooth wear, and how to best care for their dentition.

*And I thought the harder you brush your teeth the better and that—I rubbed grooves all the way down sort of the sides of my teeth. I rubbed the enamel off*,* and that was the—before my Bulimia started.* (Participant 13: 47-year-old female, eating disorder aetiology)

Some participants were made aware of their tooth wear by their dentist and had tried to address this and monitor progression.

*It’s a strange one*,* because it happens so gradually that I didn’t really notice. Then my dentist started saying*,* “It looks like you’re wearing your teeth down* (Participant 10: 39-year-old male, extrinsic dietary aetiology)

Those with an aetiology of intrinsic acids from eating disorders particularly stressed the importance of raising concerns regarding wear early and the benefits of directing information toward patients regarding this.

*And*,* I went in and I was expecting him to*,* obviously notice. And he was really nice actually. He just said*,* “Have you currently got*,* or have you had a history of Bulimia?” And he was lovely. And he actually had leaflets on the regional*,* well sorry the national eating disorder charity is actually in Norwich. It’s from there*,* it’s called BEAT* (Participant 13: 47-year-old female, eating disorder aetiology)

In some situations, despite knowing the information, the difficulty in making dietary changes in line with health messages was apparent.

*I was aware of it because I had read about it and with having children…I did know really but I just ignore it. I think basically because my daughter had soft teeth when she was younger and*,* I probably looked around how I could help her teeth*,* rather than actually my own teeth. You know*,* because she had to have numerous teeth out when she was only about four.* (Participant 4: 61-year-old female, intrinsic gastric aetiology)

Difficulties in isolating the causes of tooth wear translated into challenges in communication between patients and clinicians. This led to confusion in patients regarding their diagnosis and the cause of their condition.

*because I don’t really know at the end of the day*,* why they wore down—they said I had a lot of—what… reflux—but it’s not something I really suffered with.* (Participant 5: 66-year-old male, extrinsic dietary aetiology)

*It was clear yeah*,* but on the letter it had put on like excessive use of carb—like*,* diet—like*,* pop. But I don’t have excessive use of pop.* (Participant 6: 63-year-old male, intrinsic gastric aetiology)

Often wear with tooth loss was seen as just a natural progressive age-related change with some having an acceptance of the inevitability of having false teeth. These assumptions included common medical concerns like acid reflux being normal as well.

*I’m sixty-five*,* sixty-six and I just thought teeth go along the slightly ivory yellowish side at that age was normal. I sort of thought it a bit of a blessing that I still had them.* (Participant 5: 66-year-old male, extrinsic dietary aetiology)

It was clear, where patients understood the cause and self-reflected, they were able to identify behaviour patterns in their life that were causing their wear and tried to address this.

*Yeah*,* I think the effect of alcohol obviously played a big part—and me diet at the time which is when you drink—when you’re an alcoholic you don’t have a very good diet.* (Participant 11: 67-year-old male, parafunction aetiology)

*So*,* I think… when I was younger*,* I didn’t look after them as well as I should have done. You know like*,* in my… late teens*,* twenties I was drinking a lot of fizzy drinks*,* and then in my twenties I was going out a lot*,* drinking like a lot of like*,* alcopops or*,* you know*,* the like*,* other like fizzy drinks on nights out and stuff. And then*,* when I got older*,* I think maybe I started getting like life’s stresses started to stress me out a bit more*,* and I think I was grinding my teeth at night and I didn’t realise and*,* so like so now that the lifestyle’s changed*,* I’m not really doing the—I’m not drinking the fizzy drinks as much but I think I’ve still—I’ve got like more stress in my life now so that*,* probably like in my sleep—because I do toss and turn a lot and I think I’m grinding my teeth as well so*,* yeah I think that was causing it too.* (Participant 10: 39-year-old male, extrinsic dietary aetiology)

However, it was apparent that behaviour change was particularly difficult when complicated by medical conditions. Participants with medical conditions attributed their wear to their medical condition. Those with chronic illnesses where behaviour change is required to manage aetiology reported finding it harder to manage causative factors.

*I’m also a type one diabetic*,* so I do drink a lot of Lucozade with my—and basically there was no way round that*,* if your blood sugars are low*,* you’ve got to have some form of sugar so*,* whether it was Lucozade*,* whether it was a bag of sweets*,* it was sugar*,* and there was no way round that.* (Participant 3: 46-year-old female, intrinsic gastric aetiology)

*It was just the Bulimia that’s caused the problems*,* it wasn’t negligence.* (Participant 13: 47-year-old female, eating disorder aetiology)

## Discussion

This study is one of the first to qualitatively explore the effects of tooth wear on OHRQOL, informing the development of a tooth wear-specific PROM. Thematic analysis produced three broad themes and underlying sub-themes which encompass a broad range of impacts from a diverse sample with lived experience of tooth wear. These impacts can directly inform the development of a much-needed tooth wear-specific PROM, which will have a multitude of applications both in clinical care and in research. To the authors knowledge, this is the first study to qualitatively explore the impact of tooth wear to generate items for a condition-specific PROM.

This study has demonstrated that people with tooth wear have varied experiences. with mental, physical and emotional impacts of their condition. The effect of tooth wear on a person’s appearance, daily function and interaction with others has been well documented in this study and emotive language was often used by participants to describe their feelings. A study utilising OHIP-26 to measure the impact of OHRQoL among adult patients found that there was low satisfaction in tooth wear patients regarding functional limitation, physical discomfort and psychological discomfort [5]. This aligns with the some of the findings of the present study regarding participant’s self-esteem and pain symptoms such as sensitivity.

Participants in the present study with varied tooth wear severity used emotive language to describe the impact of their condition on their lives. Efforts have been made to quantify the relationship between OHRQoL and the clinical severity of tooth wear [5, 23]. One study reported a positive correlation between increasing Basic Erosive Wear Examination (BEWE) scores in patients with tooth wear and their responses to the Oral Impact on Daily Performance (OIDP) instrument, a generic measure of OHRQoL [24]. This suggests a decreasing oral health-related quality of life in patients, as the severity of tooth wear increases. It should be noted that, although originally developed for erosive tooth wear, [25] the BEWE instrument has subsequently been found to be a useful tool for assessing tooth wear of multi-factorial aetiology [26]. Interestingly, this relationship between tooth wear severity and OHRQoL has been found to be less notable in a general population, rather than a patient sample. A secondary analysis of data from the Adult Dental Health Survey showed a small significant association between tooth wear and OHRQoL, in those with severe tooth wear only [27]. 

The impact of tooth wear on oral health-related quality of life in the aforementioned studies has been measured using a range of generic instruments, which makes it challenging to compare the findings [28]. The standardised use of a condition-specific instrument for tooth wear as part of a core outcome set, would facilitate easy comparison and synthesis of the results to gain clarity on the national, and international picture for this condition. There is currently no available condition-specific measure of OHRQoL for tooth wear. A previous modification to the OIDP has been made, [12, 24] which generates a separate condition-specific score from a subset of questions relating to tooth wear, yet it should be noted that this is not a self-administered instrument, and is based on the socio-dental concept of estimating treatment need, rather than specifically measuring OHRQoL [29]. Furthermore, the condition-specific scoring system of OIDP requires longitudinal validation to determine sensitivity to change in impacts over time, and following treatment provision [30]. Whilst generic instruments are able to capture these impacts to a degree, condition-specific instruments have been found to be more responsive in measuring health related quality of life, with higher levels of sensitivity to changes in quality of life following treatment [13]. As such, a condition-specific PROM for tooth wear is likely to be able to provide greater detail regarding the impacts of tooth wear, and enhanced sensitivity to detect changes in OHRQoL over time, including during and after treatment for tooth wear.

There are several strengths to this study. The purposive sampling approach enabled this study to gain perspectives from a broad range of participants, which allowed a breadth of tooth wear experiences from varying aetiology, severity and stage of treatment journey, to be explored. This was supported by the use of a sampling strategy, which ensured that the data reflected the complexity, variation and depth of participants experiences of tooth wear. With regards to data saturation, it should be noted that no new codes were created from the last two transcripts. However, the inclusion of the final two participants was necessary to meet the requirements of our sampling strategy, and demonstrates the authors efforts to capture the views of individuals from a range of ages, genders and ethnicities.

This study also has a number of limitations. All participants were current or past patients of a dental hospital where patients receive free dental care, and were identified by their normal clinical care team, which may have led to courtesy bias. There was a gender imbalance, with 76.5% of participants identifying as male. This however reflects the prevalence of tooth wear, which is known to affect males more than females [21, 22]. 

This study has generated a large pool of items that could be used in a PROM for tooth wear. Further research is required to develop this PROM, including item reduction, questionnaire design, testing of the psychometric properties [14]. Once developed and implemented, the PROM could have many uses, including evaluation of treatment outcomes in terms of improvement in OHRQoL, rather than reliance on clinical parameters for success alone. Widespread use of this PROM could also help inform commissioning decisions.

## Conclusion

This is the first study to qualitatively demonstrate the broad impacts that tooth wear can have on patients’ oral health-related quality of life and has generated a wealth of items to inform the development of a novel measure of OHRQoL specific to tooth wear. Once developed and implemented, this condition-specific PROM can help to guide clinical management and aid commissioning of services for tooth wear.

## Supplementary Information

Below is the link to the electronic supplementary material.

Supplementary Material 1

## Data Availability

Anonymised datasets for this study are available upon reasonable request to the authors.

## Declarations

### Author information

Nil.
