# Supplementary figures and images for: Item generation for a tooth wear-specific patient-reported outcome measure
Source: Health Qual Life Outcomes. 2026 Mar 17;24:53. doi: 10.1186/s12955-025-02472-x (PMC13107632; doi:10.1186/s12955-025-02472-x)

**
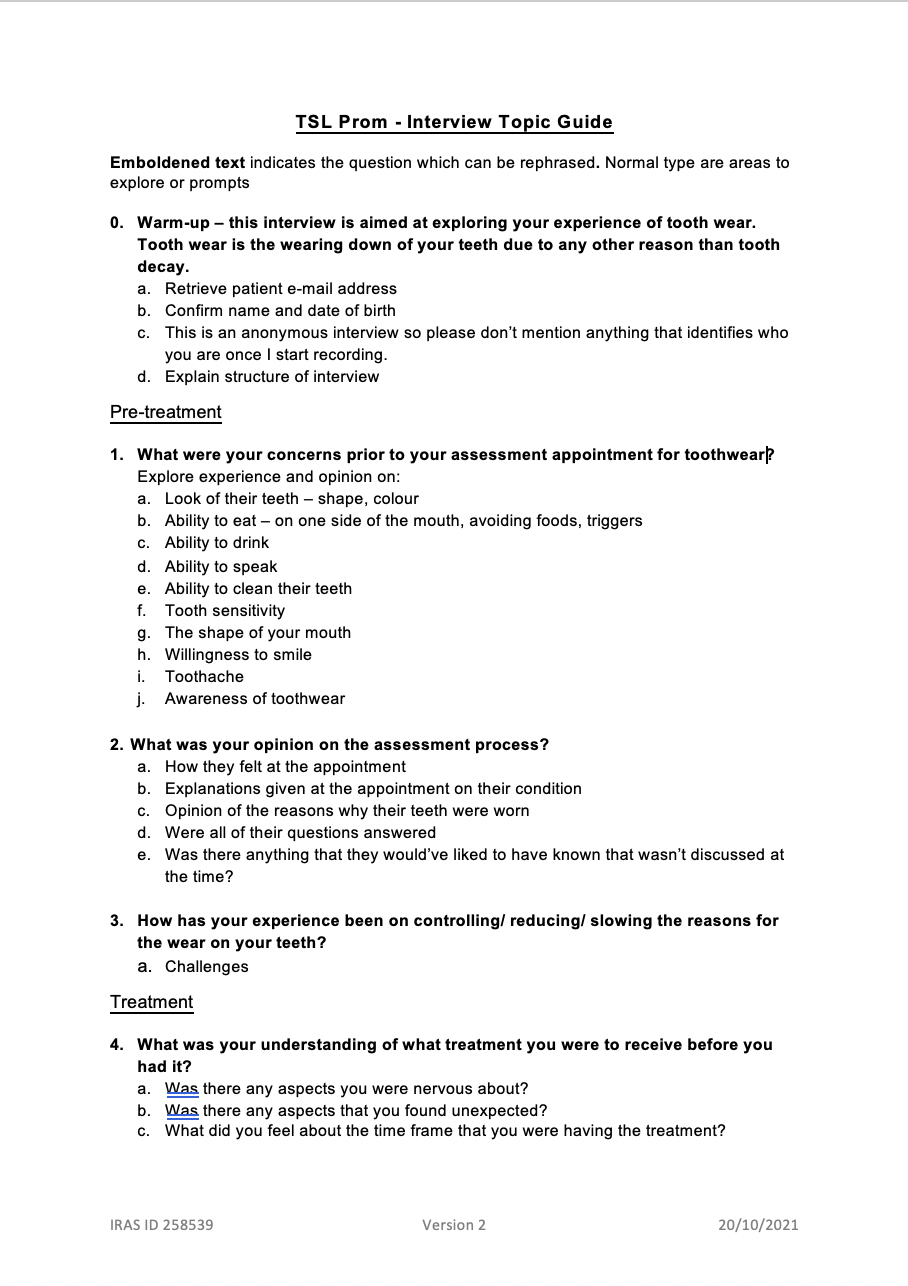
**

**
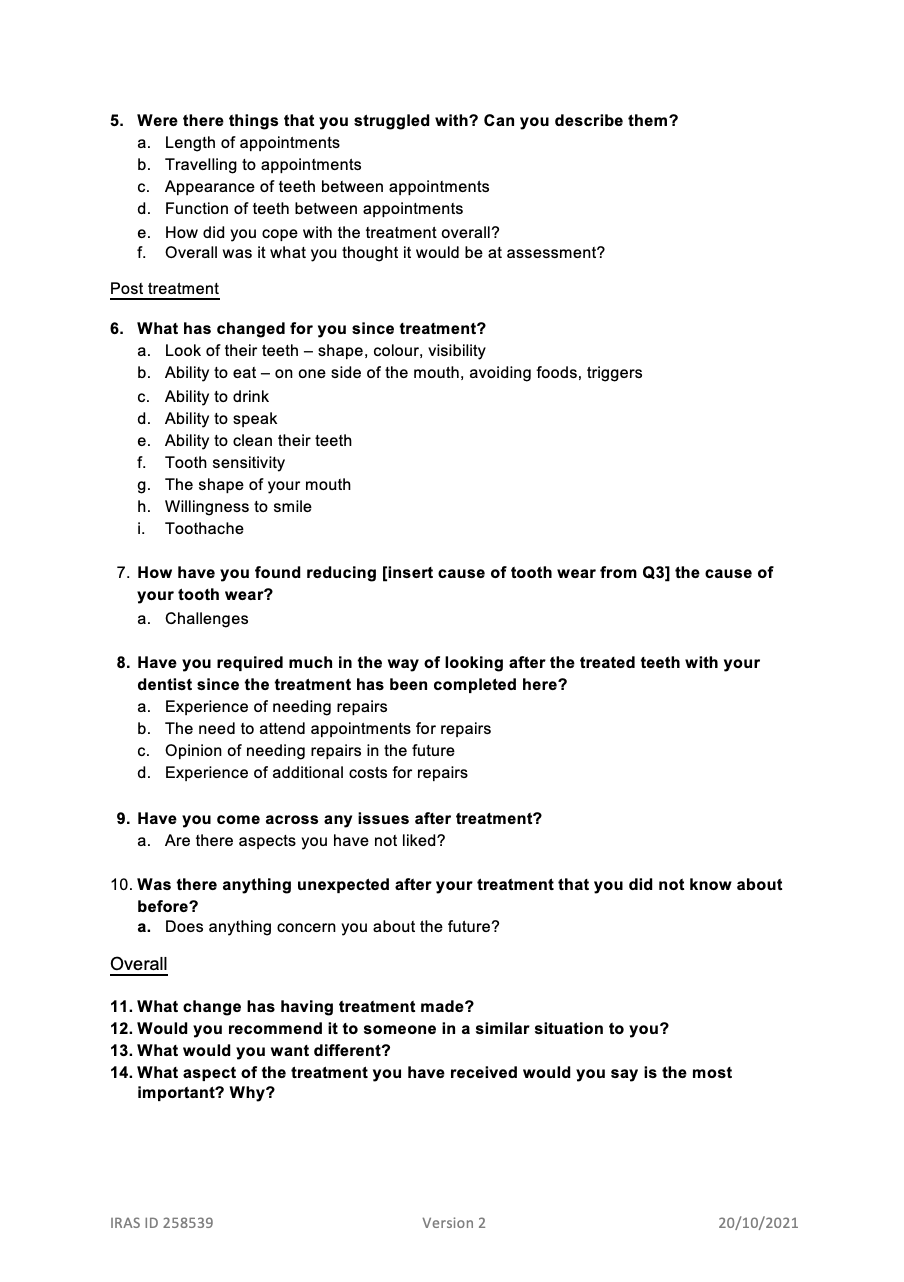
**

Supplement: Supplementary file 1 — Supplementary Material 1 [file 12955_2025_2472_MOESM1_ESM.docx]
